# Diverse Papillomavirus Types Induce Endosomal Tubulation

**DOI:** 10.3389/fcimb.2019.00175

**Published:** 2019-05-28

**Authors:** Abida Siddiqa, Paola Massimi, David Pim, Lawrence Banks

**Affiliations:** ^1^Tumour Virology Laboratory, International Centre for Genetic Engineering and Biotechnology, Trieste, Italy; ^2^Department of Microbiology and Immunology, Center for Molecular Tumor Virology, Feist-Weiller Cancer Center, Louisiana State University (LSU) Health Shreveport, Shreveport, LA, United States

**Keywords:** papillomavirus, infectious entry, MICAL-L1, endosomal tubulation, PV trafficking

## Abstract

Previous studies have shown that the endoplasmic reticulum (ER)-anchored protein VAP is strictly required by human papillomavirus type 16 (HPV-16) for successful infectious entry. Entry appeared to be mediated in part through the induction of endosomal tubulation and subsequent transport of the virion to the trans-Golgi network (TGN). In this study, we were interested in investigating whether this mechanism of infectious entry is conserved across multiple Papillomavirus types. To do this, we analyzed the role of VAP and endosomal tubulation following infection with Pseudovirions (PsVs) derived from the alpha, beta, delta, kappa, and pi papillomavirus genera, reflecting viruses that are important human and animal pathogens. We demonstrate that VAP is essential for infection with all PV types analyzed. Furthermore, we find that VAP and EGFR-dependent endosomal tubulation is also induced by all these different Papillomaviruses. These results indicate an evolutionarily conserved requirement for VAP-induced endocytic tubulation during Papillomavirus infectious entry.

## Introduction

The Papillomavirus family comprises over 200 different virus types, certain of which are responsible for the development of epithelial tumors and cancer (Steben and Duarte-Franco, 2007). The L1 and L2 capsid proteins of Papillomaviruses play essential roles in the establishment of infection. The L1 protein facilitates virus attachment to the extracellular matrix (Johnson et al., 2009), which initiates conformational changes in the viral capsid that help in uptake of the infectious particle through endocytosis (Yang et al., 2003; Selinka et al., 2007; Day et al., 2008), with the internalization of Papillomaviruses believed to be dependent on EGFR signaling (Schelhaas et al., 2012; Surviladze et al., 2012, 2013). The viral capsid undergoes disassembly due to endosomal acidification, resulting in exposure of the L2 protein. The L2/DNA complex then separates from the majority of the L1 with the help of cellular cyclophilins (Bienkowska-Haba et al., 2012), and most of the L1 protein is then degraded in the lysosome (Buck et al., 2013). A portion of the L2 protein spans the endosomal membrane during this process and recruits cellular sorting factors, including members of the sorting nexin protein family, components of the retromer, retriever and the ESCRT complex (Bergant Marusic et al., 2012; Broniarczyk et al., 2014; Pim et al., 2015; Popa et al., 2015; McNally et al., 2017), all of which facilitate the trafficking of the L2/vDNA complex, together with a small amount of L1, toward the trans-Golgi network (TGN) (Day et al., 2013; DiGiuseppe et al., 2017). The L2/vDNA complex is believed to reside in the TGN until the cell undergoes mitosis. The events of membrane dissolution and nuclear envelope breakdown during mitosis then allow the L1/L2/vDNA complex to enter the nucleus and to accumulate at PML oncogenic domains (PODs), where the initiation of viral gene expression is believed to take place (Day et al., 2004; Pyeon et al., 2009; Aydin et al., 2014; DiGiuseppe et al., 2017).

The early endosome, as it matures, forms tubular extensions that are believed to play a role in cargo sorting and recycling. These tubules detach from the endosome and traffic cargoes to the Golgi complex or recycle them back to the plasma membrane (Huotari and Helenius, 2011; Gautreau et al., 2014). Another important aspect of endocytic trafficking is the establishment of contact points between the endosome and the endoplasmic reticulum (ER), which are critical for cargo trafficking from the endosome to the TGN (Dong et al., 2016). The VAMP (vesicle-associated membrane protein)-associated protein (VAP) is thought to be important in making endosome-ER contact points, since depletion of VAP results in phosphatidylinositol 4-phosphate (PI4P) accumulation in the endosome, and dysfunctioning of the retromer and Wiskott-Aldrich Syndrome Protein and SCAR Homolog complex (WASH), leading to disruption of cargo trafficking from the endosome to the TGN (Dong et al., 2016). We have recently shown that VAP is important for HPV-16 PsV infectious entry and, moreover, HPV-16 PsV induces VAP-dependent endosomal tubulation, without which the incoming virus is unable to reach the TGN (Siddiqa et al., 2018). In this study we were therefore interested in investigating whether induction of tubulation was common to different Papillomavirus types, and, furthermore, whether the activity of VAP at endosomal-ER contact points was also required for infectious entry of multiple PV types.

## Methods

### Cell Culture

HeLa cervical cancer cells, VAP Double Knockout (DKO) HeLa cells [generated using TALEN-based gene editing to abolish the expression of VAP-A and VAP-B; kindly provided by Pietro de Camilli (Dong et al., 2016)], and HEK293TT human embryonic kidney cells were grown in Dulbecco's modified Eagle's medium (DMEM) containing 10% fetal calf serum (FBS), penicillin streptomycin (100 U/ml), and glutamine (300 μg/ml).

To confirm the absence of VAP expression in the VAP DKO HeLa cells, the cells were harvested and lysed in E1A buffer. Cell lysates were then analyzed by western blot for levels of VAP-B expression in WT and VAP DKO HeLa cells.

### Plasmids, Antibodies, and Inhibitor

The following plasmids were used to make PsVs: p16shell.L2-3xFLAG-thrombin-HA (Zhang et al., 2014); pV18cap (Campos et al., 2012) kindly provided by Samuel Campos; pV2-31LLh (Smith et al., 2007) kindly provided by Michelle Ozbun; p2shell (Cerqueira et al., 2017), p5shell (Buck et al., 2006), pBPVshell (Buck et al., 2004), pCRPVshell (Roberts et al., 2007), and pMushell (Handisurya et al., 2012), all kindly provided by Christopher Buck. They carry bicistronic sequences encoding the L1 and L2 capsid proteins from HPV-16, HPV-18, HPV-31, HPV-2, HPV-5, BPV-1, SfPV-1, and MmuPV-1 respectively. The plasmid pGL3 luci, which carries the firefly[108mm][-12mm] Q10 luciferase gene, was purchased from Promega.

Mouse anti-MICAL-L1 (Novus Biologicals), rabbit anti-α-actinin (Santa Cruz), mouse anti-VAP-B (Abcam) and mouse anti-pERK1/2 antibody (Cell Signaling) were used for immunofluorescence or western blotting. The EGFR-specific inhibitor PD168393 was purchased from Sigma-Aldrich.

### Pseudovirion Production and Labeling

HPV-16, HPV-18, HPV-31, HPV-2, HPV-5, BPV-1, SfPV-1, and MmuPV-1 PsVs with a packaged luciferase reporter gene (pGL3 luci) were generated in HEK293TT cells as described previously (Buck et al., 2005). The purity of the PsVs samples, was determined by SDS-PAGE analysis. The quantitation of packaged pGL3 DNA for viral genome equivalent (vge) was carried out by real-time PCR, using a standard curve of reporter plasmid DNA. For EdU labeling, growth medium was supplemented with 25 μM EdU at 12 h post-transfection during PsV production. All PsVs were used in equivalent amounts to those of HPV-16 PsVs.

### Infectivity Assays

Wild type (WT) HeLa and VAP DKO cells were infected with diverse PsVs for 48 h at a multiplicity of infection (m.o.i.) of ~50 vge/cell. Infectivity was monitored by measuring the firefly luciferase activity using a luciferase assay system kit (Promega).

To check the role of EGFR signaling in infection, HeLa cells were treated with 300 nM of the EGFR-specific inhibitor PD168393 for 30 min, or with DMSO as a control, prior to infection with 50 vge/cell. The inhibitor was maintained during the 48 h of infection and cell viability was >90%. The luciferase activity was monitored as a measure of infectivity 48 h post-infection, as described above.

Twenty four hours after inhibitor application, cells were treated with 10 ng/ml of EGF for 15 min to confirm that the EGFR signaling is perturbed in response to the inhibitor. Cell lysates were then analyzed by western blot for levels of pERK1/2 expression.

### PsVs Trafficking Assay

WT HeLa and VAP DKO cells (2.5 × 10^5^ per well) were seeded in 6-well plates. Cells were infected with PsVs at 150 vge/cell, together with EdU-labeled reporter DNA, and agitated at 4°C for 1 h to allow virus attachment to the cells. Cells were then washed with phosphate-buffered saline (PBS), supplemented with DMEM, and incubated at 37°C for 2, 8, and 24 h post-infection. Cells were fixed with 3.7% paraformaldehyde for 15 min at room temperature. Immunofluorescence for MICAL-L1 was performed as described before (Siddiqa et al., 2018). Images were obtained using a Zeiss Axiovert 100 M microscope, and analyzed by using an LSM image browser that supports the LSM 510 confocal unit.

### Data Analysis

The mean data from three independent experiments was analyzed and plotted using GraphPad Prism 6. Standard error was determined, and statistical significance was sought through one way ANOVA or Student's *t*-test. The *p*-value below 0.05 was considered statistically significant and throughout, the *p*-values have been defined as follows ^*^p < 0.01, ^**^p < 0.001, ^****^p < 0.00001. Briefly, to check the infectivity of PsVs under different conditions, relative luminescence was measured for three independent experiments. For quantification of EdU labeled reporter DNA in WT HeLa and VAP DKO infected cells, at least 150 cells for 2 h post-infection from three independent experiments for each cell line were analyzed. EdU particles were manually counted and percentage was calculated using total cell numbers and the data for VAP DKO cells were normalized to the WT HeLa for each PsV type. The number of MICAL-L1 positive tubules in WT HeLa and VAP DKO cells were manually counted and percentage was calculated using total number of cells analyzed for UI, 2, 8, and 24 h post-infection, respectively. At least 150 cells under each condition from three independent experiments were analyzed if stated otherwise. The data for WT HeLa was normalized to the tubulation observed in UI cells for each PsVs. The data for VAP DKO HeLa cells was normalized to tubulation observed in WT HeLa for each PsVs at specific time points, respectively.

## Results

### VAP Is Required for Infectious Entry With Different Papillomavirus Types

**Figure 1:**
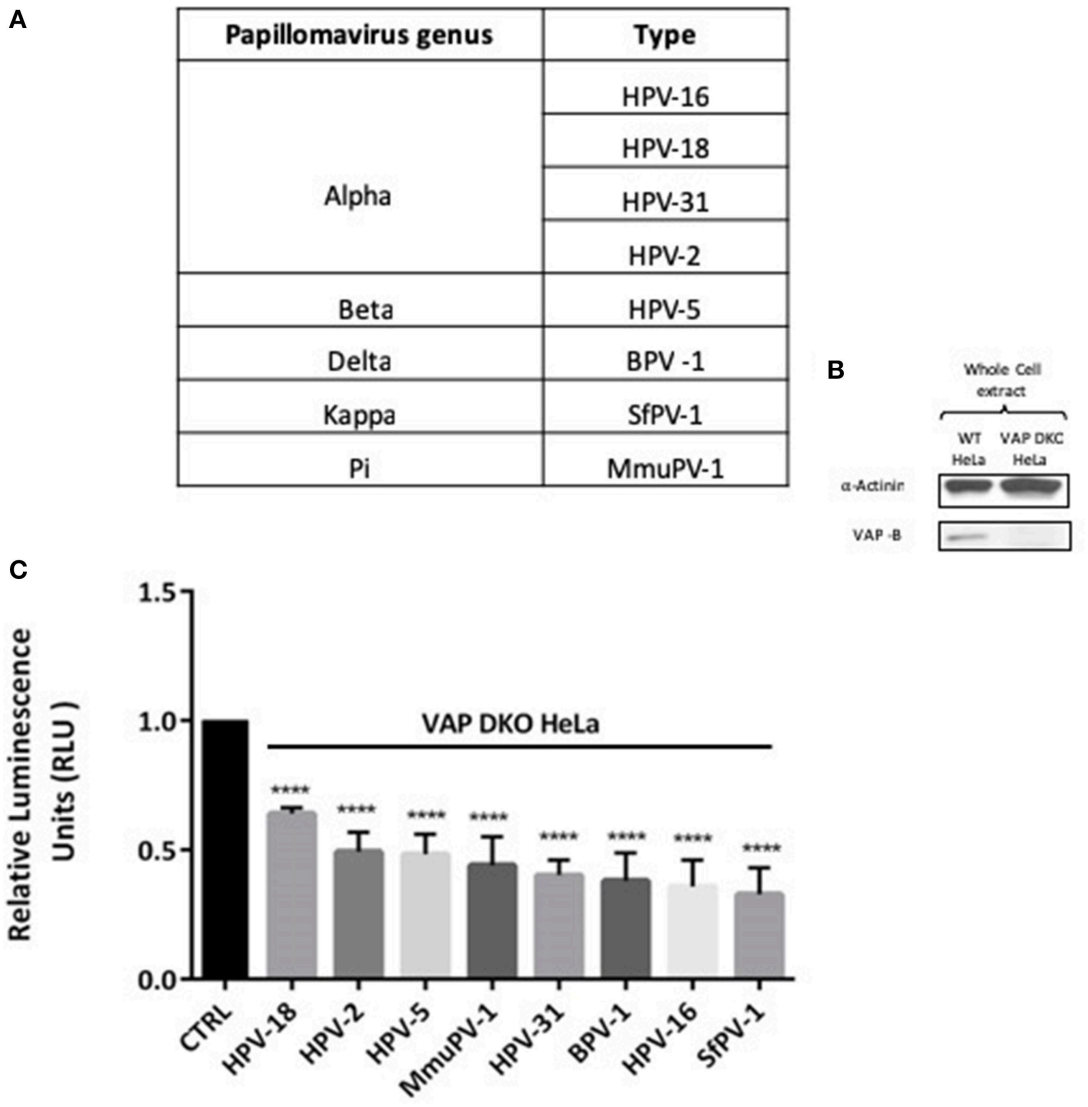
Infection by diverse papillomavirus types depends on the integral ER protein VAP. **(A)** List of different PVs analyzed in this study. **(B)** Western blot shows the efficacy of VAP-B knockdown. **(C)** WT HeLa (CTRL) and VAP DKO HeLa cells were infected with the indicated PVs for 48 h. Relative luminescence was measured and is plotted as a bar graph. The data shown here are the mean luciferase readings derived from 3 independent experiments, normalized against the respective infection in WT HeLa, where the bars indicate standard errors. Significance was determined using one way ANOVA (^****^P < 0.00001).

In order to investigate the requirement of VAP for infectious entry with different Papillomavirus types, we performed a series of infection experiments using representative PsVs derived from genus alpha (HPV-18, HPV-31, HPV-2), genus beta (HPV-5), genus delta (BPV-1), genus kappa (SfPV-1), and genus pi (MmuPV-1), all of which carried a luciferase reporter construct and are enlisted in Figure 1A. The loss of VAP-B in the VAP DKO HeLa cells is confirmed by western blotting (Figure 1B). WT and VAP DKO HeLa cells were infected with the different PsVs and, after 48 h, the cells were harvested and luciferase activity was measured. All PsVs were used in equivalent amounts to those of HPV-16 PsVs (50 vge/cell). For each PsV the luciferase activity obtained in VAP DKO HeLa cells is normalized to the respective luciferase activity in WT HeLa cells. The results in Figure 1C show a significant decrease in the infectious entry of all these PV types (*P* < 0.00001; one-way ANOVA) when VAP expression is knocked down, indicating that the VAP requirement for infectious entry of Papillomaviruses is evolutionarily highly conserved.

### Role of VAP in Inducing Endosomal Tubulation With Diverse Papillomavirus Types

**Figure 2:**
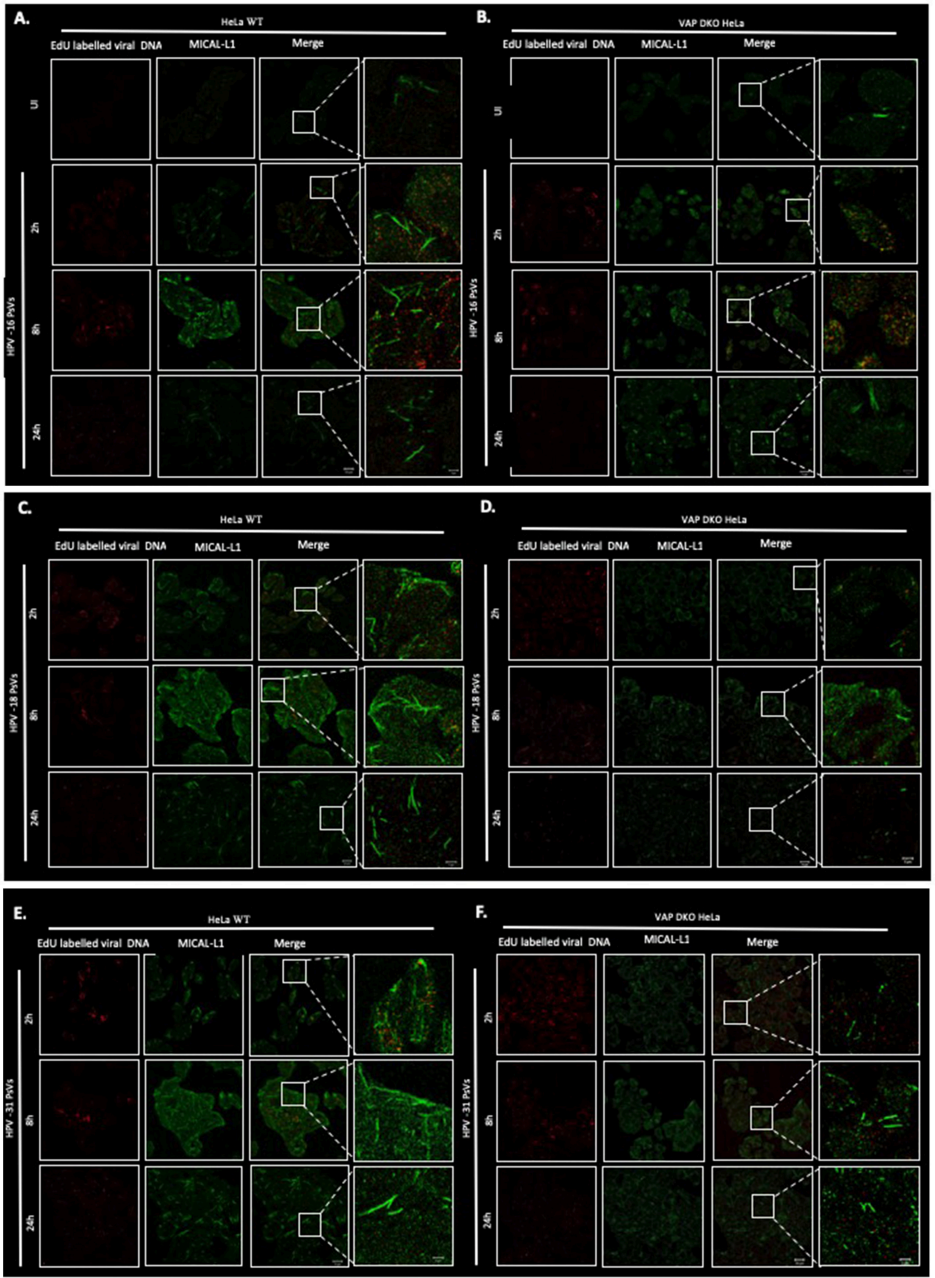
Representative images of multiple PV types inducing VAP-dependent endosomal tubulation. WT HeLa **(A)** and VAP KDO HeLa **(B)** cells were infected with HPV-16 PsVs (150 vge/cell), and fixed at 2, 8, and 24 h post-infection. Reporter DNA encapsidated within the PsVs is detected by EdU labeling (red), whereas endogenous MICAL-L1 is stained with MICAL-L1 antibody (green) as a marker of an endosomal tubulation. Experiments are performed at least three times. Images were captured by confocal microscopy. The same analysis was performed with HPV-18 PsVs in WT HeLa **(C)** and in VAP KDO HeLa **(D)** cells; HPV-31 PsVs in WT HeLa **(E)** and in VAP KDO HeLa **(F)** cells; BPV-1 PsVs in WT HeLa **(G)** and in VAP KDO HeLa **(H)** cells; MmuPV-1 PsVs in WT HeLa **(I)** and in VAP KDO HeLa **(J)** cells. Scale bar: 20 μm. The right-hand column shows the zoomed images. Scale bar: 5 μm.

**Figure 3:**
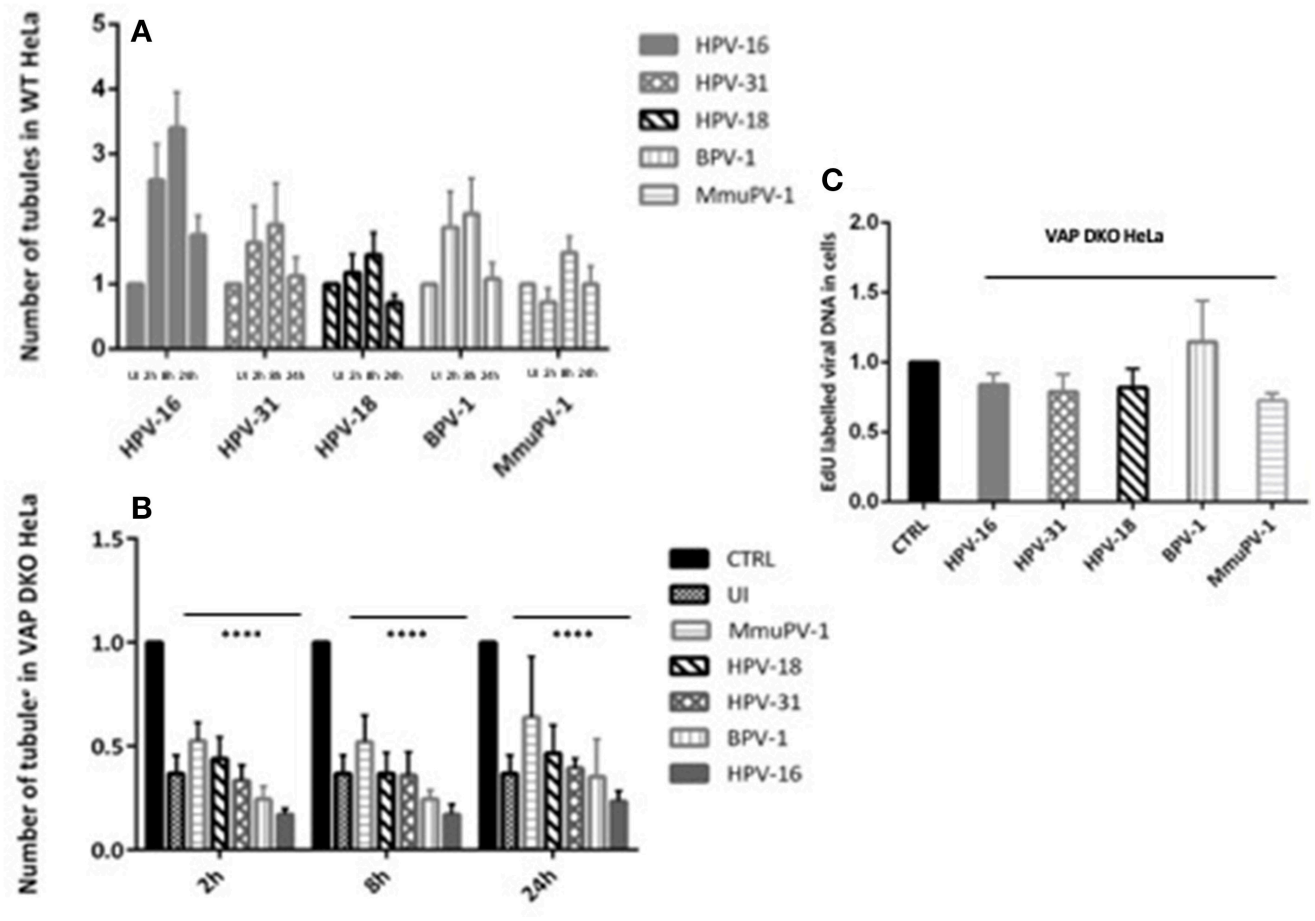
The number of tubules and EdU labeled viral DNA in WT HeLa and VAP KDO HeLa cells was quantified. At least 150 cells under each condition from three independent experiments were analyzed. **(A)** Number of endosomal tubules in WT HeLa cells were counted for UI, 2, 8, and 24 h post-infected cells and data was normalized to the uninfected cells. The data shown here is the mean number of tubules. An increase in tubulation was observed for all PV types analyzed: the increase observable as early as 2 h, becoming maximum at 8 h with a reduction at 24 h post-infection. Bars indicate standard errors. **(B)** Number of endosomal tubules in VAP-DKO HeLa cells were counted and data was normalized to the number of tubules counted in the WT HeLa for each respective PsV type (CTRL). Data shown here is the mean number of tubules. The dramatic decrease in tubulation in the absence of VAP is significant as found by Student's *t*-test comparing UI, 2, 8, 24 h WT with UI, 2, 8, 24 h VAP DKO HeLa cells for each PsVs, respectively (^****^P < 0.00001). Bars indicate the standard error **(C)** EdU labeled viral DNA in VAP-DKO HeLa cells was counted for 2 h post-infection and data was normalized to the EdU counted in the WT HeLa for each PsV type (CTRL). The data shown here is the mean number of EdU particles. There is very slight difference in EdU in the absence of VAP. Bars indicate the standard error.

After establishing that VAP is required for the efficient infectious entry of these diverse Papillomavirus types, we were interested to know whether VAP also has a role in inducing endosomal tubulation, as was observed previously for HPV-16 infection (Siddiqa et al., 2018). WT and VAP DKO HeLa cells were infected with diverse EdU-labeled PsVs. All PsVs were used in equivalent amounts to those of HPV-16 PsVs (150 vge/cell), and tubulation was analyzed 2, 8, and 24 h post-infection, using immunofluorescence staining for molecules interacting with CasL-like 1 (MICAL-L1), which is widely used as a marker of endosomal tubulation. HPV-16 PsVs were used as a control and, as can be seen from Figure 2, the maximum increase in endosomal tubulation was observed by 8 h post-infection in WT HeLa (Figure 2A). In contrast, endosomal tubulation was greatly reduced in VAP DKO HeLa cells (Figure 2B), which is consistent with previous studies (Siddiqa et al., 2018). We extended this analysis to other members of genus alpha (HPV-18, HPV-31), to genus delta (BPV-1), and to genus pi (MmuPV-1), to determine whether they also induce endosomal tubulation in a VAP-dependent manner. As can be seen from Figures 2C–J, **3A**, all the Papillomavirus types analyzed induced endosomal tubulation as early as 2 h post-infection in WT HeLa cells. This increased at 8 h post-infection and was reduced by 24 h post-infection, a pattern of endosomal tubulation very similar to that seen with HPV-16 PsVs. In contrast, when the same assays were performed in VAP DKO HeLa cells this tubulation was largely absent, confirming that the requirement for VAP in the induction of endosomal tubulation is conserved across multiple Papillomavirus types. The endosomal tubulation for each PsV in WT HeLa cells are quantified from three independent experiments and plotted in a bar graph (Figure 3A). The data was normalized to the tubulation observed in the uninfected cells. The endosomal tubulation in VAP DKO HeLa cells was also quantified, as shown in Figure 3B. The data was normalized with respect to the tubulation observed in WT HeLa cells for each virus, respectively. As shown in Figure 3B, the loss of endosomal tubulation is statistically significant (*P* < 0.00001; Student's *t*-test) across the range of PsVs. In order to show that loss of tubulation in VAP DKO HeLa cells is not due to differences in the uptake of viruses, EdU was counted in WT and VAP DKO HeLa cells from three independent experiments at 2 h post-infection and shown in Figure 3C. Only a very slight difference in EdU was observed in VAP DKO cells in comparison to the WT HeLa. In our previous study we have mentioned that tubulation was induced in HeLa cells even when virus was titrated down to 30 vge/cell. This suggests that tubulation can occur with very less amount of virus, and its loss in VAP DKO cells is more likely VAP dependent.

### EGFR Signaling Is Important for Infectious Entry and Endosomal Tubulation

**Figure 4:**
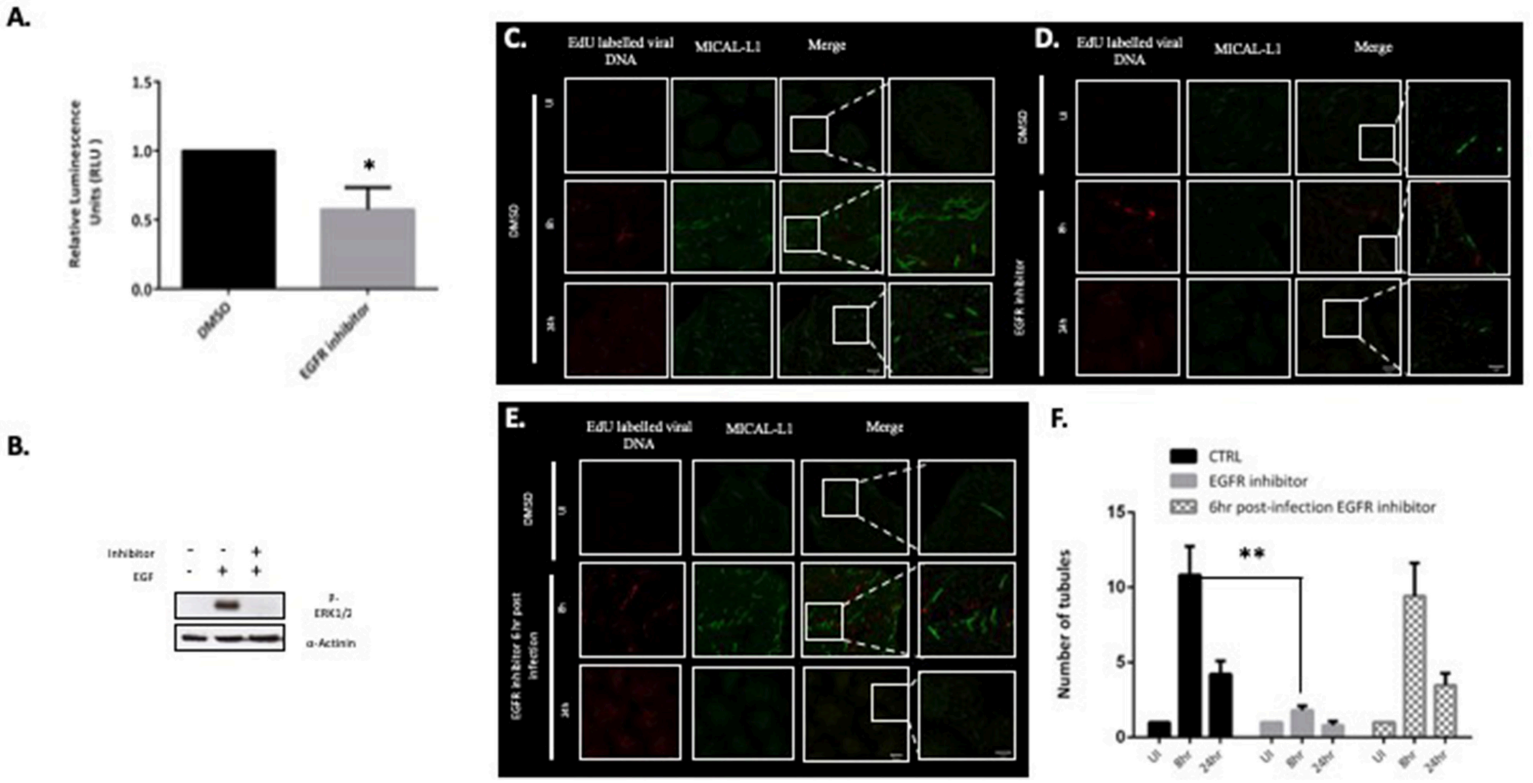
EGFR signaling is required for virus uptake and endocytic tubulation. **(A)** HeLa cells were treated with 300 nM of the EGFR-specific inhibitor (PD168393). DMSO-treated cells were used as a control. Cells were infected with HPV-16 PsVs (50 vge/cell) carrying a luciferase reporter plasmid and luciferase activity was measured at 48 h posttransduction. The data shown are the mean luciferase readings from three independent experiments, where bars indicate standard errors. Significance was determined using Student's *t*-test (^*^P < 0.01). **(B)** Western blot for p-ERK1/2 following 10 min EGF (10 ng/ml) exposure in the presence or absence of PD168393 was performed in HeLa cells. **(C)** HeLa cells were treated with DMSO and infected with HPV-16 PsVs (150 vge/cell) for 8 and 24 h. Tubulation was detected with MICAL-L1 (green). **(D)** HeLa cells were treated with 300 nM PD168393 and infected with HPV-16 PsVs (150 vge/cell) for 8 and 24 h. **(E)** HeLa cells were infected with HPV-16 PsVs (150 vge/cell) and treated with 300 nM PD168393 6 h post- infection. Scale bar: 10 μm. The right-hand column shows the zoomed images. Scale bar 5 μm. **(F)** The number of tubules in HPV-16 PsVs infected HeLa cells (CTRL), EGFR inhibitor treated cells and 6 h post-infection EGFR inhibitor treated cells were quantified. At least 50 cells under each condition (UI, 8 and 24 h infection) from three independent experiments were analyzed and data was normalized to the uninfected cells. Data shown here is the mean number of tubules. The dramatic decrease in tubulation in the absence of EGFR signaling is significant as found by Student's *t* test comparing 8 h untreated to inhibitor treated cells (^**^P < 0.001). Bars indicate the standard error.

Previous studies have shown that loss of MICAL-L1 perturbs EGFR recycling back to the plasma membrane (Abou-Zeid et al., 2011), and EGFR signaling has also been shown to play an important role in the infectious entry of HPV (Surviladze et al., 2012). We therefore hypothesized that EGFR signaling might also be playing a role in Papillomavirus-induced tubulation, as this would indicate a requirement for endocytic uptake of the incoming virus for tubulation to occur. To examine this possibility, HeLa cells were first treated with 300 nM of the EGFR-specific inhibitor PD168393. Efficacy of the inhibition was ascertained by measuring pERK1/2 by western blotting (Figure 4B). HeLa cells were infected with the HPV-16 PsVs and, as can be seen from Figure 4A, treatment with inhibitor resulted in a dramatic decrease in HPV-16 infectious entry, which is consistent with previously published studies (Surviladze et al., 2012). The role of EGFR signaling in endosomal tubulation was then ascertained by treating HeLa cells with PD16893, and then infecting them with EdU-labeled HPV-16 PsVs. Cells were then fixed and stained for MICAL-L1 at 8 h and 24 h post-infection. As can be seen from Figure 4D, inhibition of EGFR signalling resulted in dramatic decrease in endosomal tubulation in comparison to control in Figure 4C. Having shown that tubulation is lost in the absence of EGFR signaling, we were next interested in determining whether tubulation would be perturbed if EGFR signaling was abolished once infection had already been established. Cells were infected with HPV-16 PsVs and treated with EGFR-specific inhibitor at 6 h post-infection. As can be seen from Figure 4E, inhibition of EGFR signaling post-infection had no effect on endosomal tubulation. The data from three independent experiments is quantified and plotted in a bar graph (Figure 4F). The data was normalized to the tubulation observed in the uninfected cells. The loss of endosomal tubulation in presence of EGFR inhibitor is statistically significant (*P* < 0.001; Student's *t*-test).

This suggests that EGFR signaling is required in the early stages of virus infection, most likely promoting virus endocytosis, which is in turn required for endosomal tubulation. However, once the virions have been endocytosed, the subsequent abrogation of EGFR signaling does not affect the endosomal tubulation process.

## Discussion

Endocytic trafficking plays an essential role in the successful infectious entry of HPV-16, as it allows the transport of viral cargo from endosome to TGN, from whence it subsequently enters the nucleus. We had previously shown the induction of endosomal tubulation in response to HPV-16 PsVs infection, as indicated by MICAL-L1 imaging (Siddiqa et al., 2018). However, these events of endosomal tubulation depend upon the ER-associated VAP protein, without which the incoming viral DNA/L2 fails to reach the TGN and instead remains trapped in Vps29-TGN46 hybrid vesicle structures. Our previous study has shown that in the absence of VAP, HPV-16 infection is not blocked before capsid uncoating, indicating that infectious virus entry is not blocked from the beginning (Siddiqa et al., 2018). In the current study we have shown that VAP protein is essential for the infectious entry of diverse Papillomavirus genera. This in turn is also required for virus-induced endosomal tubulation and suggests that ER-endosome contact points are important for trafficking the incoming viruses to the TGN. Moreover, EGFR signaling plays an essential role in this process at the early stages of virus infection, indicating that viral endocytosis is required for the induction of endosomal tubulation. However, it needs to be emphasized that some Papillomavirus infectious entry still occurs despite the complete loss of VAP in the VAP DKO cells. This suggests that Papillomaviruses most likely make use of multiple entry pathways, in which there are substantial elements of redundancy. This has been shown in many similar studies, where loss of a particular trafficking component does not necessarily block infectious entry completely but results in a marked reduction in the efficiency of infection (Bergant Marusic et al., 2012; Lipovsky et al., 2013; Cerqueira et al., 2015; Pim et al., 2015; Grassel et al., 2016).

VAP provides the contact point between the ER and endosome. One aspect of these contact points is that they facilitate endosomal fission: when cargo-containing endosomal tubules or vesicles come into contact with the ER, they are marked by the retromer-associated protein FAM-21, which defines the time and position of endosomal fission (Rowland et al., 2014). It has been suggested that endosome maturation and trafficking is coupled to ER contact points. Interestingly, these contact points increase in number as the endsome matures (Friedman et al., 2013). It is likely that VAP-dependent ER-endosome contact points facilitate the ER-mediated cleavage of virion-positive vesicles, which then reach the TGN. Further studies are needed to fully understand how the incoming virions affect tubulation, and how diverse papillomaviruses use this system to their advantage.

MICAL-L1 is frequently used as a marker of endosomal tubulation (Sharma et al., 2009; Giridharan et al., 2013; Compeer and Boes, 2014; Etoh and Fukuda, 2019). Its precise function is not known, however it has been shown to play a role in recycling certain cargoes, including transferrin and EGFR, from the elongated tubular endosomal network (ETEN) and late endosomes toward the plasma membrane (Compeer and Boes, 2014). The presence of HPV-16 positive MICAL-L1 tubules (Siddiqa et al., 2018) indicates that ETEN might play a role in tubules destined for the TGN, as well as in those trafficking to the plasma membrane. The evidence suggesting a role for MICAL-L1 in EGFR recycling is further strengthened by another study that shows the F-BAR protein PACSIN2 (an alternative marker for endosomal tubulation) is involved in the regulation of EGFR signaling (Kreuk et al., 2012). Our study shows loss of endosomal tubulation when EGFR signaling is blocked, pointing to the role of EGFR signaling both in infection and in virus-induced endosomal tubulation. However, more work is required to validate the precise mechanism involved.

Taken together, these results demonstrate a highly conserved role for endosomal-ER contact in infection with multiple Papillomavirus types, and suggest a highly conserved pathway of endosomal trafficking, via ER-associated vesicular processing, to ensure viral entry into the TGN.

## Author Contributions

AS, LB, DP, and PM designed the experiments and analyzed the data. AS performed the experiments. AS and LB wrote the manuscript.

### Conflict of Interest Statement

The authors declare that the research was conducted in the absence of any commercial or financial relationships that could be construed as a potential conflict of interest.
